# Small molecule inhibitors reveal allosteric regulation of USP14 via steric blockade

**DOI:** 10.1038/s41422-018-0091-x

**Published:** 2018-09-25

**Authors:** Yiwei Wang, Yuxuan Jiang, Shan Ding, Jiawang Li, Ningjing Song, Yujing Ren, Danning Hong, Cai Wu, Bin Li, Feng Wang, Wei He, Jiawei Wang, Ziqing Mei

**Affiliations:** 1 Biotechnology Research Institute, Chinese Academy of Agricultural Sciences, Beijing, 100081 China; 2 Tsinghua-Peking Center for Life Sciences, School of Life Sciences, Tsinghua University, Beijing, 100084 China; 3 School of Pharmaceutical Sciences, Tsinghua University, Beijing, 100084 China; 4 School of Life Science, Beijing Institute of Technology, Beijing, 100081 China; 5 School of Life Sciences, Anhui Agricultural University, Hefei, Anhui 230026 China

## Abstract

The ubiquitin system is important for drug discovery, and the discovery of selective small-molecule inhibitors of deubiquitinating enzymes (DUBs) remains an active yet extremely challenging task. With a few exceptions, previously developed inhibitors have been found to bind the evolutionarily conserved catalytic centers of DUBs, resulting in poor selectivity. The small molecule IU1 was the first-ever specific inhibitor identified and exhibited surprisingly excellent selectivity for USP14 over other DUBs. However, the molecular mechanism for this selectivity was elusive. Herein, we report the high-resolution co-crystal structures of the catalytic domain of USP14 bound to IU1 and three IU1 derivatives. All the structures of these complexes indicate that IU1 and its analogs bind to a previously unknown steric binding site in USP14, thus blocking the access of the C-terminus of ubiquitin to the active site of USP14 and abrogating USP14 activity. Importantly, this steric site in USP14 is very unique, as suggested by structural alignments of USP14 with several known DUB X-ray structures. These results, in conjunction with biochemical characterization, indicate a coherent steric blockade mechanism for USP14 inhibition by compounds of the IU series. In light of the recent report of steric blockade of USP7 by FT671, this work suggests a potential generally applicable allosteric mechanism for the regulation of DUBs via steric blockade, as showcased by our discovery of IU1-248 which is 10-fold more potent than IU1.

## Introduction

Ubiquitination is one of the most versatile post-translational modifications in eukaryotic cells, dictating the fates of proteins by linking different types of polyubiquitin chains.^1,2^ The ubiquitin system is expected to furnish as many drug targets as the phosphorylation system, given the profound complexity of this system and its ample associations with various important diseases.^3^ However, there have been few successful drug discovery efforts focused on the ubiquitin system, partly due to the lack of useful small-molecule tool compounds.

The difficulties associated with compound discovery are especially pronounced in the case of inhibitors of deubiquitinating enzymes (DUBs).^3^ Considerable efforts have been dedicated to the discovery of small molecules that functionally inhibit DUBs.^4^ Due to the low druggability and highly conserved nature of DUBs, previous efforts have mainly focused on covalent inhibitors, i.e., compounds that form covalent bonds with the active site cysteines.^5,6^ These compounds usually have poor selectivity across the DUB family.^4,7,8^ In 2010, Finley, King and coworkers reported the first-ever specific inhibitor targeting DUBs, namely, IU1, targeting USP14.^7^ As IU1 was also proposed to be an active site-directed thiol protease inhibitor, the selectivity of IU1 was especially surprising and puzzling.^7^ Very recently, several non-covalent inhibitors, including XL188, compound 4 and FT671, were reported.^5,8,9^ Due to their allosteric regulatory mechanisms, these compounds exhibited very high selectivity for USP7 among the DUB family. These two different explanations (covalent for USP14 versus allosteric for USP7) for the compound selectivity suggested that a consensus on the understanding of the selectivity is currently lacking.

It is in this context that we set out to understand the molecular basis of the selectivity of IU1. We hope to reconcile the different views regarding compound selectivity, which might, in the long run, facilitate DUB drug discovery.^10^ Herein, we solved the co-crystal structures of USP14 with IU1 and three other IU1 derivatives at atomic resolution. We also characterized the binding mode of IU1 to USP14. The results showed that IU1 exerted its inhibitory activity by binding to the thumb-palm cleft region of the USP14 catalytic domain, which prevented the binding of the C-terminus of ubiquitin to USP14 via steric blockade. Based on the structures, we designed and synthesized IU1-248, an IU1 derivative that is 10-fold more potent than IU1. In conjunction with previous findings from a study of the binding of FT671 with USP7, the results of this work suggest that allosteric regulation via steric blockade might be a viable approach for DUB inhibitor discovery. Our findings also provide valuable information for structure-guided design of steric blockade inhibitors, considering that rational compound design was not an option until very recently^5,9^ even though the apo structures of USP14 and USP7 had been solved for many years.^11,12^

## Results

### Characterization of inhibition of USP14 hydrolysis by IU1

**Fig. 1:**
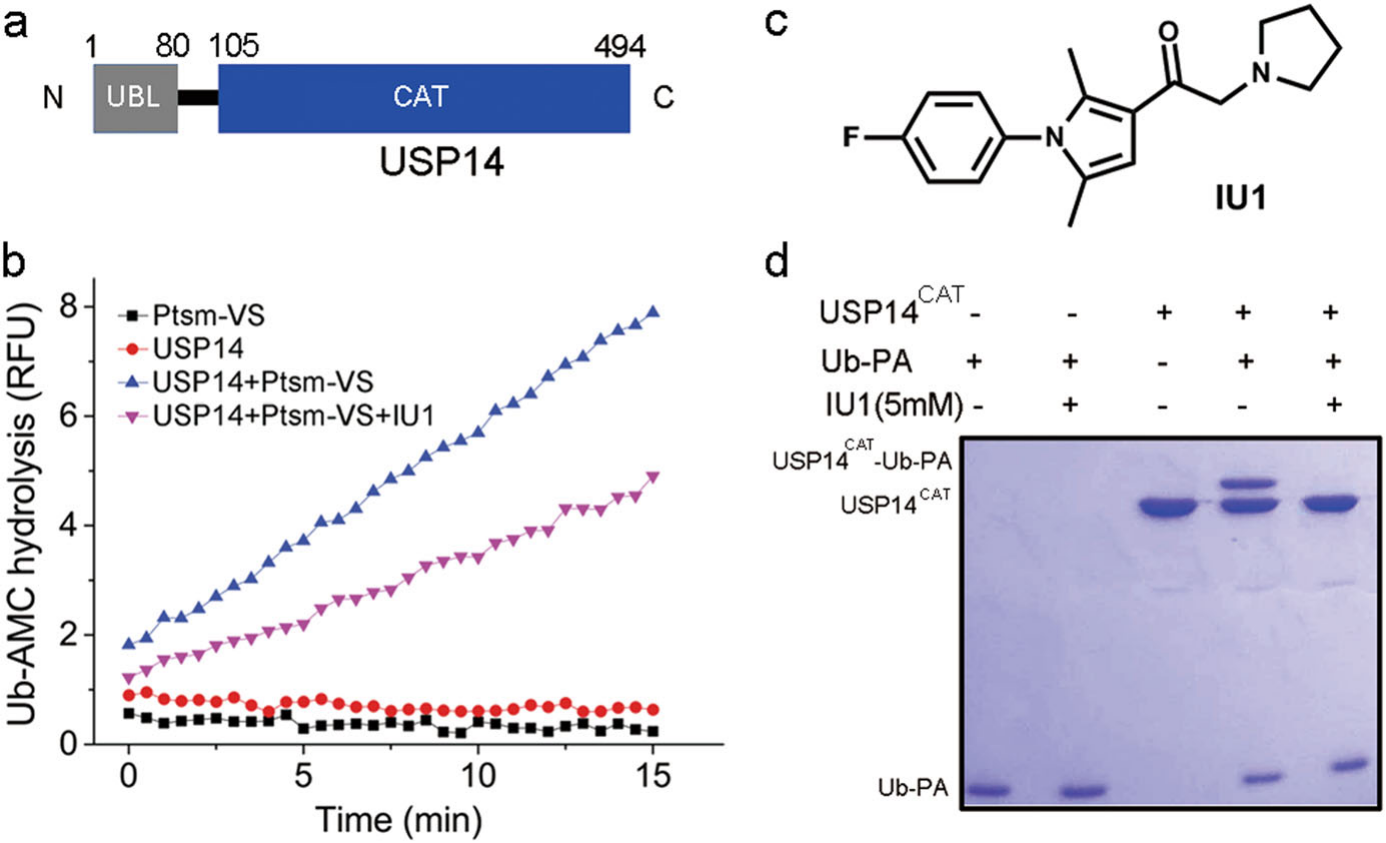
Characterization of USP14 and IU1. **a** Domain structure of human USP14. USP14 comprises two domains: an N-terminal ubiquitin-like domain (UBL, 1-80) and a C-terminal catalytic domain (CAT, 105-494). **b** IU1 inhibited the Ub-AMC hydrolysis activity of proteasome-activated USP14: 15 μM IU1 exhibited detectable inhibition of 15 nM USP14 activated by 1 nM proteasome. Ub-AMC ubiquitin-7-amino-4-methylcoumarin, Ptsm-VS proteasome inhibited by ubiquitin-vinyl sulfone (Ub-VS). **c** Chemical structure of IU1. **d** The Ub-PA assay suggests that IU1 inhibits USP14 activity by preventing substrate binding: 3 μM USP14^CAT^ was preincubated with DMSO or 5 mM IU1 for 1 h at 25 °C and then mixed with 12 μM Ub-PA for 1 h. All the results were visualized by SDS-PAGE and Coomassie blue staining. Ub-PA ubiquitin-propargylamide

Full-length human USP14 contains 494 amino acids and consists of two structural domains: an N-terminal ubiquitin-like domain (UBL, 1–80) and a C-terminal catalytic domain (CAT, 96–494) (Fig. 1a).^12^ USP14 alone is autoinhibited by two loops, namely, BL1 and BL2. The protein is activated upon incorporation with the 26S proteasome or upon phosphorylation.^13–17^ IU1 is the first reported noncovalent selective inhibitor towards USP14 by Finley, King and coworkers.^7^ To evaluate the mechanism by which IU1 targets USP14, we reconstituted the deubiquitinating activity of USP14 as previously described.^7^ We purified the 26S proteasome treated with ubiquitin-vinyl sulfone (Ptsm-VS), which exhibited undetectable DUB activity but retained the ability to activate USP14 (Fig. 1b). As expected, the recombinant USP14 did not exhibit any Ub-AMC (ubiquitin-7-amino-4-methylcoumarin) hydrolysis activity but could be activated by Ptsm-VS. The synthesized IU1 exhibited equivalent inhibition of Ub-AMC hydrolysis by USP14^7^ (Fig. 1b, c).

As the binding affinity between IU1 and USP14 had not been reported, we tried several biophysical methods including ITC (isothermal titration calorimetry), SPR (surface plasmon resonance) and MST (microscale thermophoresis), which all failed due to the poor solubility and the low affinity of IU1.^7,18^ We then turned to the biochemical Ub-PA (ubiquitin-propargylamide) assay. In this assay, the catalytic domain of USP14 (USP14^CAT^) was preincubated with IU1, followed by addition of the covalent inhibitor Ub-PA.^19–22^ The covalent attachment of Ub-PA (8 kD) to USP14^CAT^ (45 kD) produced the novel USP14/Ub-PA complex with an increased molecular weight (53 kD). We observed that at a 5 mM concentration IU1 significantly inhibited the formation of the USP14/Ub-PA complex (Fig. 1d), which is in good agreement with the proposed steric blockade mechanism (vide infra).

### Overall structure of human USP14^CAT^ bound to IU1

**Fig. 2:**
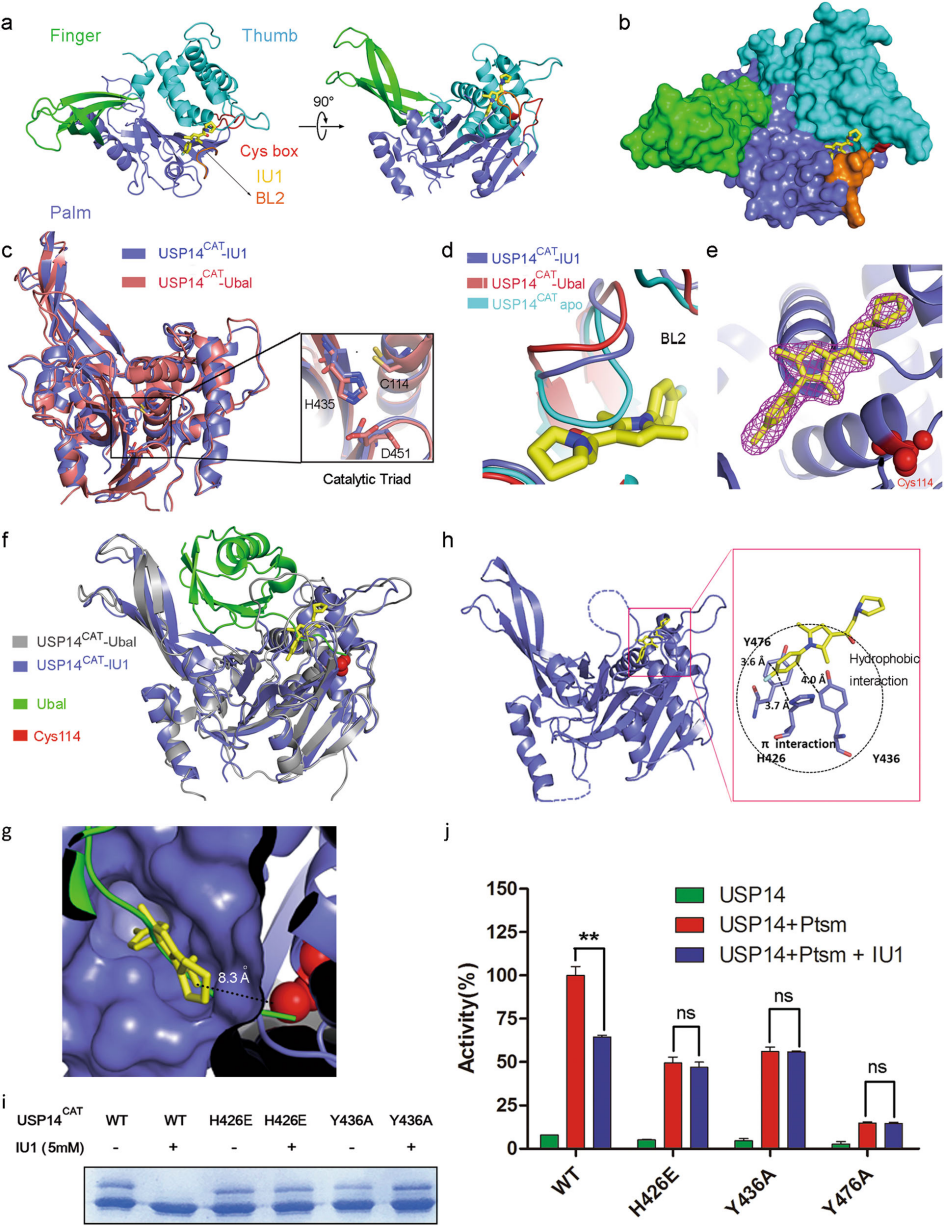
Overall structure of human USP14^CAT^-IU1 and recognition of IU1 by USP14. **a** Overall structure of the USP14^CAT^-IU1 complex. USP14^CAT^ comprises finger (green), palm (slate) and thumb (cyan) domains. The crucial surface loop BL2 is shown in orange. IU1 (yellow) binds the cleft between the palm and thumb domains. The catalytic center (Cys box) is shown in red. **b** Surface representation of the structure of the USP14^CAT^-IU1 complex. **c** Structural alignment of USP14^CAT^-IU1 and USP14^CAT^-Ubal (PDB ID: 2AYO) shows that these complexes share a similar structure. The catalytic triad residues Cys114, His435, and Asp451 are magnified. Neither IU1 nor Ubal is shown in the figure. **d** Structural alignment of BL2 in apo USP14^CAT^, USP14^CAT^-Ubal and USP14^CAT^-IU1. BL2 in USP14^CAT^-IU1 exhibits a similar position as that in USP14^CAT^-Ubal. **e** 2|Fo| − |Fc| electron density maps, contoured at 1.5 σ and covering all atoms of IU1. The catalytic center Cys114 is shown as red spheres. **f** Alignment of the structure of USP14^CAT^-IU1 with that of USP14^CAT^-Ubal shows that IU1 works blocks the access of the C terminus of ubiquitin to the catalytic center. Ubal is colored green. **g** The binding pocket for IU1 (yellow) in USP14 (slate). IU1 is 8.3 Å away from the catalytic center (red). **h** Recognition of IU1 by USP14. IU1 interacts with H426, Y436 and Y476 of USP14 via hydrophobic interactions and π-π stacking (π–π interactions). USP14 is colored in slate, and IU1 is colored in yellow. **i** H426 and Y436 are involved in IU1 recognition, as proved by the Ub-PA assay. The H426E or Y436A mutation rescued the Ub-PA covalent binding activity of USP14 upon the addition of 5 mM IU1. **j** Key residues involved in IU1 recognition were sequentially verified by an Ub-AMC hydrolysis assay: 15 μM IU1 significantly inhibited USP14 WT activity but exhibited no inhibition of the USP14 H426E, Y436A and Y476A mutants. All the values correspond to the averages of triplicate experiments, and the error bars represent SDs. Double asterisks denote *p* < 0.01. ns not significant

To investigate the molecular basis of the selectivity of IU1 toward USP14, crystallization trials for the complex of USP14^CAT^ bound to IU1 (USP14^CAT^-IU1) were carried out. Due to the limited binding affinity, the co-crystals of human USP14^CAT^-IU1 were difficult to obtain. After numerous trials, the co-crystal structure of USP14^CAT^-IU1 at a high resolution (1.96 Å; Fig. 2a, b) was successfully solved by molecular replacement using the atomic model of human USP14 (PDB ID: 2AYN). Data collection and refinement statistics are given in Table 1.

**Table 1:** Data collection and refinement statistics

	USP14-IU1	USP14-IU1-47	USP14-IU1-206	USP14-IU1-248
Space group	P2_1_	P2_1_	P2_1_	P2_1_2_1_2_1_
Unit cell (Å, ˚)	57.869 81.67 107.371	58.427 81.404 108.161	58.31 81.186 108.511	81.809 104.351 118.271
Unit cell (˚)	90, 93.68, 90	90, 94.624, 90	90, 94.741, 90	90, 90, 90
Resolution (Å)	50.00–1.96 (2.03–1.96)	40.00–2.20 (2.28–2.20)	30.00–2.21 (2.29-2.21)	36.25–2.53 (2.62–2.53)
<*I*/*σI*>	28.25 (2.32)	33.05 (3.95)	19.46 (3.41)	16.93 (3.14)
Completeness (%)	96.71	93.66	95.89	98
Redundancy	3.4 (3.3)	4.3 (3.4)	3.1 (2.8)	5.5 (5.8)
Wilson B factor	36.57	45.48	41.77	49.9
No. of reflections	68,318	48,528	48,730	33,924
*R*_work_/*R*_free_	0.1885/0.2181	0.1875/0.2317	0.2062/0.2469	0.2105/0.2670
No. of atoms				
Protein	5388	5429	5390	5413
Ligand	44	46	48	50
Water	322	183	274	104
B factor				
Protein	46.86	49.49	55.31	58.85
Ligand	45.25	53.36	50.55	57.93
Water	41.53	35.86	40.77	42.83
R.M.S. deviations				
Bond length (Å)	0.008	0.008	0.008	0.003
Bond angles (˚)	1.208	1.221	1.08	0.75

The architecture of USP14 in the co-crystal structure was similar to that in the USP14-Ubal complex (PDB ID: 2AYO), with an RMSD of 1.2 Å for 288 aligned backbone Cα atoms. The catalytic triad residues Cys114, His435, and Asp451 in the two structures could be superimposed with an RMSD of 0.1 Å (Fig. 2c). The structure of USP14^CAT^-IU1 also revealed key conformational changes due to IU1 binding. The crucial surface loop BL2 (residues 429–433) for substrate access was open in our structure, in contrast to that in the USP14 apo structure (the autoinhibited form of USP14), exhibiting a nearly identical topology as that in the USP14-Ubal complex (the active form of USP14) (Fig. 2d). IU1 binds underneath the BL2 loop with distinct electron density in the substrate-binding cleft, 8.3 Å away from the catalytic cysteine Cys114 (Fig. 2e, g).

Interestingly, structural superimposition of USP14^CAT^-IU1 with USP14-Ubal revealed that IU1 blocks the entrance of the thumb-palm cleft that guides the ubiquitin C-terminus towards the catalytic center (Fig. 2f, g). We further solved the co-crystal structures of USP14^CAT^ bound to the previously reported^18^ IU1-47, as well as two new analogues designed by us (IU1-206 and IU1-248) (Table 1). These structures showed a consistent binding mode among the IU1-type compounds. Superimposition of all the co-crystal structures showed that all these IU1 analogs were associated with steric binding in the same pocket of USP14 (Supplementary information, Fig. S1). For IU1 the sake of clarity, only the USP14^CAT^-IU1 structure has been analyzed in the following sections.

### Recognition of IU1

In the structure of USP14^CAT^-IU1, His426, Tyr436, and Tyr476 in USP14 are involved in the contacts formed by USP14 with the benzene ring of IU1, probably via both hydrophobic interactions and π-π stacking (Fig. 2h, Supplementary information, Fig. S2). Notably, the orientation of the benzene ring of IU1 is locked by the two methyl groups on the pyrrole ring (the benzene ring is perpendicular to the pyrrole ring), such that interaction of the benzene ring with His426, Tyr436, and Tyr476 is enforced. Consistent with this observation, the two methyl groups on the pyrrole ring are essential for retention of the activity of IU1-type compounds, suggesting that the π-π stacking is delicate and indispensable.

The structure-guided mutational analysis also confirmed the crucial role of the π–π stacking for the binding of IU1 to USP14. The Y436A mutant retained part of the covalent Ub-PA binding ability of the wild type and could not be inhibited by 5 mM IU1 (Fig. 2i).

In contrast, either the point mutation H426A or Y476A led to a dramatic loss of binding ability and could not be used for evaluation for IU1 recognition. The USP14^CAT^-Ubal structure showed that H426 of USP14 is involved in the recognition of ubiquitin by USP14 via the formation of a hydrogen bond with Arg74 of Ubal. A H426A mutation may abolish this H bond and abrogate the interaction between Ub and USP14 (Supplementary information, Fig. S3a). To obtain H426 mutants that retain Ub recognition but lack the ability to bind IU1, we designed the USP14 mutant H426E, whose side chain is sufficiently long for the formation of an H bond with Arg74 of Ubal. As expected, H426E retained the ability to bind Ub-PA but could not be inhibited by IU1, indicating the key role of H426 in the recognition of IU1 (Fig. 2i). Y476 may maintain the ideal architecture of the catalytic center for Ub binding via the formation of an H bond with D199 in USP14 (Supplementary information, Fig. S3a). Following the logic in the analysis of H426 above, Y476K and Y476R were evaluated by Ub-PA-based experiments. Consequently, boththese Y476 mutants could not form complex with Ub-PA, and the D199A mutant also abolished the interaction between USP14 and Ub-PA (Supplementary information, Fig. S3b).

The Ub-AMC hydrolysis assay further supported the importance of the residues mentioned above for the inhibition of USP14 by IU1. The point mutations H426E and Y436A significantly weakened the inhibitory activity of IU1 (Fig. 2j, Supplementary information, Fig. S3c–f).

### Molecular basis of the selectivity of IU1 towards USP14

**Fig. 3:**
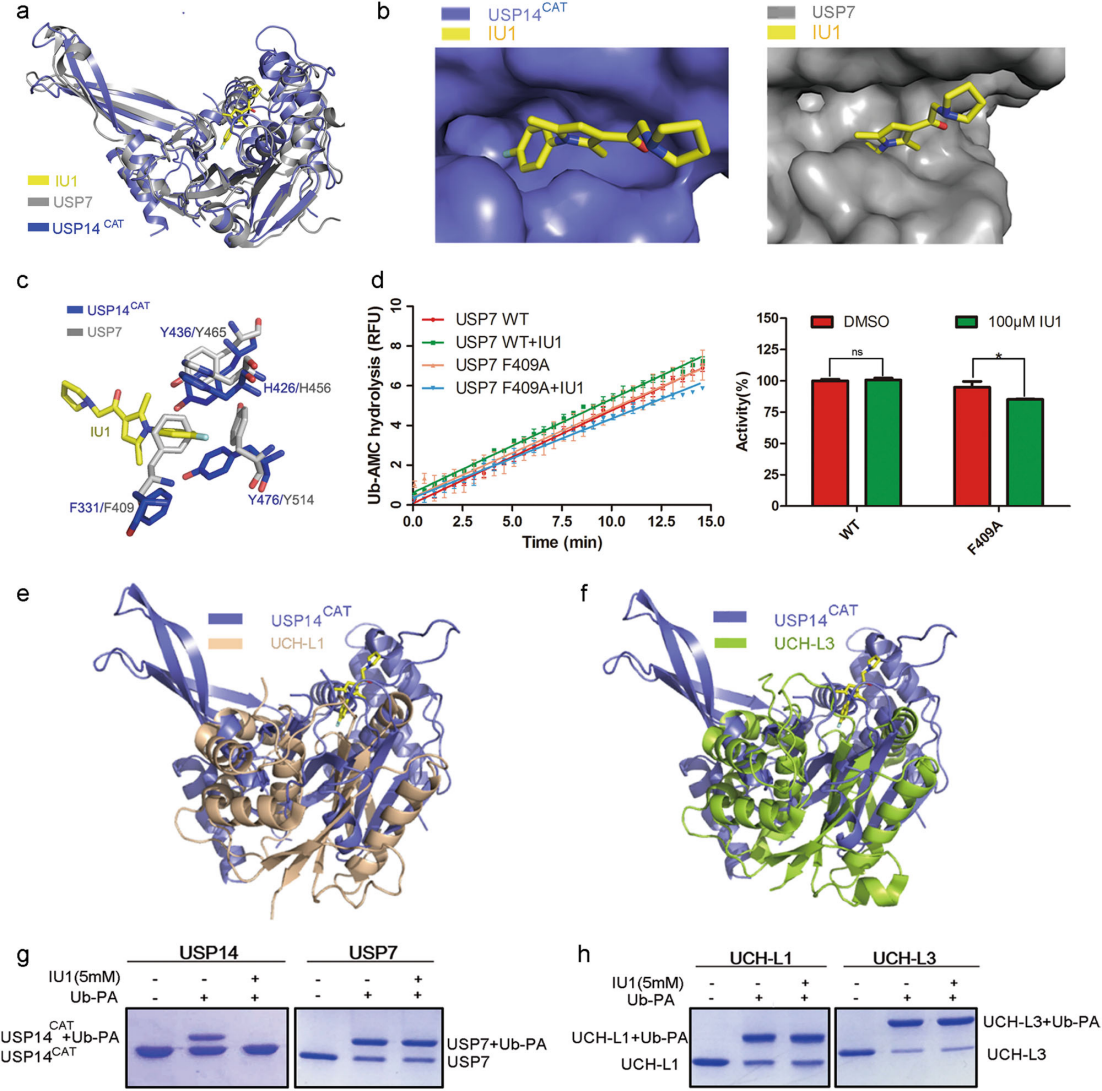
Structural alignment of the USP14^CAT^-IU1 complex with other DUB proteins. **a** USP14^CAT^-IU1 was aligned with apo USP7 (gray, PDB ID: 1NB8). **b** IU1 binding site in USP7 and USP14. **c** Comparison of the IU1 binding pockets of USP7 and USP14^CAT^. **d** 100 μM IU1 decreased the Ub-AMC hydrolysis activity of F409A but not WT USP7 protein. Linear kinetics of Ub-AMC hydrolysis are shown on the left, and the results of the statistical analysis are shown on the right. All the values correspond to the averages of triplicate experiments. Error bars represent SDs. Asterisks denote *p* < 0.05. ns not significant. **e**, **f** USP14^CAT^-IU1 was aligned with UCH-L1 or UCH-L3. **g**, **h** The Ub-PA assay showed that IU1 did not inhibit the binding of Ub-PA to USP7, UCH-L1, or UCH-L3: 3 μM DUB protein was preincubated with DMSO or 5 mM IU1 for 1 h at 25 °C and then mixed with 12 μM Ub-PA for 1 h. All the results were visualized by SDS-PAGE and Coomassie blue staining

Finley, King and coworkers have reported that IU1 can inhibit USP14 with high selectivity over USP2, USP7, USP15, IsoT (USP5), UCH-L1, UCH-L3, UCH37 and BAP1.^7^ To explain the selectivity of IU1, we first compared the sequence conservation among the USP family. USP14 shares homology with USP2, IsoT, USP7, and USP15, ranging from 28% to 35%, in the catalytic domain. The key residues in USP14 for IU1 recognition, namely, H426, Y436, and Y476, are conserved among most USP proteins (Supplementary information, Fig. S4), suggesting that variance among these key residues in the primary sequence cannot account for the selectivity of IU1. Next, to investigate whether the spatial architecture in the cleft that accommodated IU1 is unique in USP14, we performed superimposition of the USP14^CAT^-IU1 complex structure with the structures of several DUBs described in Finley’s work.^7^ First, we compared the structure of USP14^CAT^-IU1 with that of apo USP7 (Fig. 3a, b). H426 and Y436 in USP14 aligned well with the corresponding residues in USP7. However, Y514 in USP7, which is the corresponding amino acid for Y476 in USP14, was distant from IU1 and did not appear to have any chance of interacting with IU1. Moreover, F409 in USP7 sterically hindered the binding of IU1, impeding IU1 recognition (Fig. 3c). This result is supported by the observation that F409A of USP7 could be inhibited by 100 μM IU1 to some extent (Fig. 3d). Consistent with this scenario, when we compared the primary sequence and structure of USP14^CAT^-IU1 with UCH-L1 and UCH-L3, neither the primary sequence nor the structure of USP14 aligned well with UCH-L1 or UCH-L3 (Fig. 3e, f). Similar to the results of structural alignment, the covalent binding of Ub-PA to USP7, UCH-L1, and UCH-L3 were not prevented by IU1 (Fig. 3g, h). All these observations suggest that a unique binding pocket for IU1 may exist in USP14 only.

To investigate the nature of the inhibitory activity of IU1 towards USP14, the kinetic parameters *V*_max_ (*V*_m_) and *K*_m_ for Ub-AMC hydrolysis by USP14 were determined in the presence of IU1. Consequently, The *K*_m_ value increased gradually while the *V*_m_ remained unchanged. (Supplementary information, Fig. S5a-b), suggesting that IU1 functions as a competitive inhibitor of USP14.^23^ The Ki (5.0 μM) derived of IU1 from this analysis is in good agreement with the observed IC_50_ (12.3 μM).

### Structure-guided optimization of IU1

**Fig. 4:**
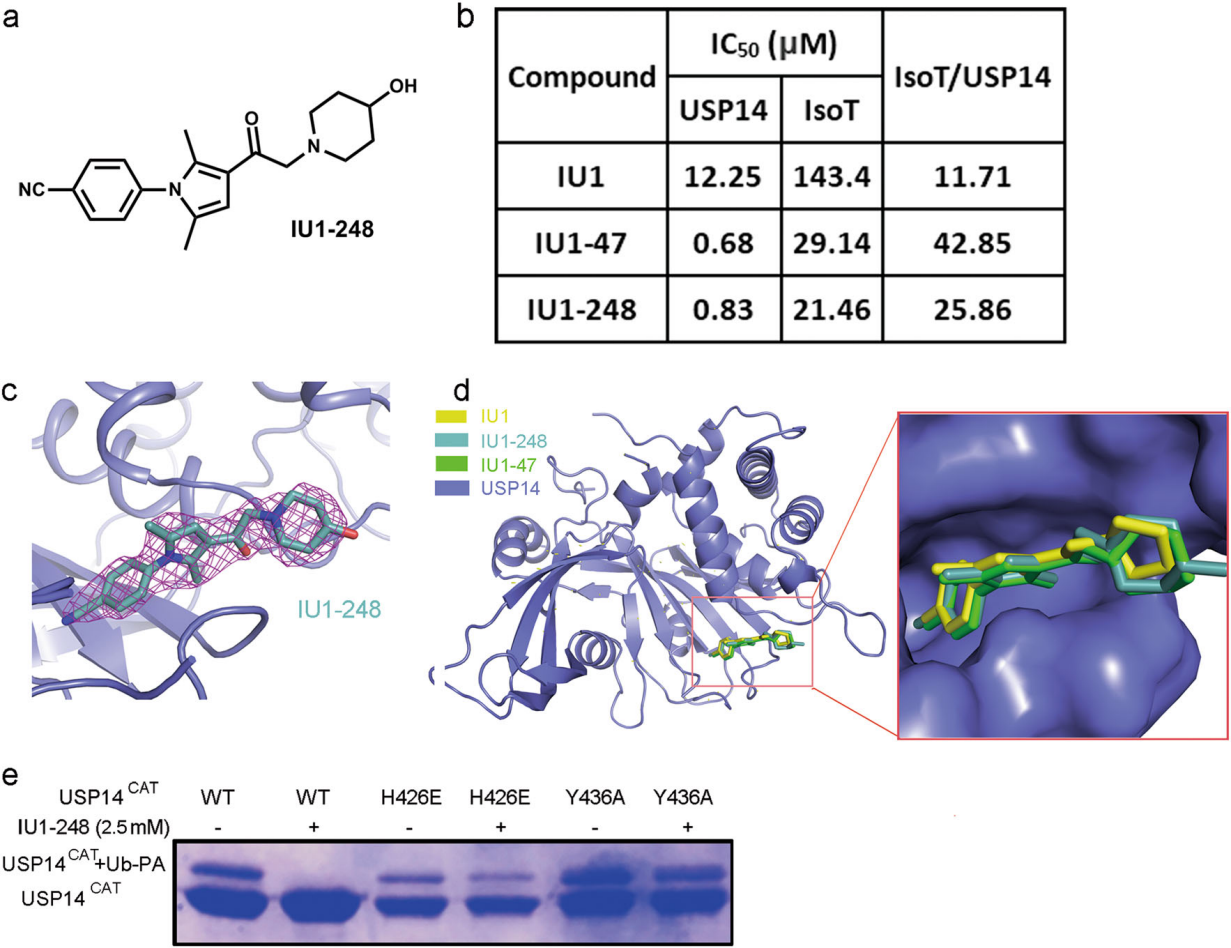
Structural-guided inhibitor design. **a** Chemical structure of IU1-248. **b** A table of the IC_50_ values of IU1 and its derivatives IU1-248 and IU1-47 toward proteasome-bound USP14 and IsoT. Similar to IU1-47, IU1-248 exhibited more than 10-fold higher potency and better selectivity than IU1. **c** 2|Fo| − |Fc| electron density maps, contoured at 1.5 σ and covering almost all the atoms of IU1-248. **d** Structural alignment of USP14^CAT^-IU1, USP14^CAT^-IU1-248 and USP14^CAT^-IU1-47 demonstrated that these inhibitors shared an identical binding pocket. **e** H426 and Y436 are involved in IU1-248 recognition, as proved by the Ub-PA assay. The H426E or Y436A mutation rescued the Ub-PA covalent binding activity of USP14 upon the addition of 2.5 mM IU1-248

Based on the structural analysis of the essential interactions between IU1 and USP14, we performed structure-guided design of new IU1-type inhibitors. Because the benzene ring and the dimethyl-substituted pyrrole ring are essential for π-π stacking, this main skeleton was maintained during inhibitor optimization. On one hand, we explored the inclusion of electron-withdrawing substituents on the benzene ring, which might enhance the π-π stacking. On the other hand, we tested several different rings to replace the pyrrolidine ring, which extended into the solvent-exposed region. Our intention was to incorporate larger rings and polar groups in order to improve the binding affinity as well as the solubility of the compounds. After several rounds of iterative optimization, we eventually discovered a compound named IU1-248 (Fig. 4a). This attractive compound had an IC_50_ value of 0.83 μM toward USP14, which is comparable to that of IU1-47 and 10-fold more potent than that of IU1 (Fig. 4b, Supplementary information, Fig. S6). Overall, both IU1-47 and IU1-248 exhibited higher selectivity for USP14 over IsoT (Fig. 4b). Notably, IU1-248 exhibited significantly improved solubility compared to that of IU1, which might be advantageous in cell-level studies.

Subsequently, we co-crystallized USP14^CAT^ with IU1-248 and determined the structure. IU1-248 exhibited apparent density in the structure (Fig. 4c); data collection and refinement statistics are given in Table 1. Alignment of USP14^CAT^-IU1, IU1-248 and IU1-47 showed that all three compounds bind in the same pocket in USP14 (Fig. 4d). Kinetic analysis suggested that IU1-248 inhibits USP14 via a competitive mechanism, similar to IU1 (Supplementary information, Fig. S5c, d). The Ub-PA assay showed that the H426E or Y436A mutation abolished the inhibitory effect of IU1-248 on USP14 (Fig. 4e).

The co-crystal structures revealed the possible reasons for the 10-fold potency gain of IU1-47 and IU1-248: (1) On the phenyl ring, the Cl- in IU1-47 and CN- in IU1-248 are larger than the F- group in IU1, filling the inner pocket of USP14 to a greater extent and providing stronger van der Waals interactions; (2) the six-membered piperidine rings of IU1-47 and IU1-248 are larger than the five-membered pyrrolidine ring of IU1, providing stronger hydrophobic interactions. We also employed molecular modeling to understand why the original compound IU1C was inactive. The modeled binding modes of all three compounds (IU1, IU1-47, and IU1-248) were consistent with those revealed by the co-crystal structures. In our modeling results, the score of the IU1C compound (−2.47) was significantly lower than those of IU1-47 (−7.11) and IU1-248 (−8.24). For IU1C, the absence of a methylene (CH_2_) linker between the ketone and the piperidine caused steric repulsion between the piperidine and the protein.

## Discussion

As a proteasome-associated DUB, USP14 has been shown to regulate numerous cellular processes, including cell cycle, DNA repair, epigenetics, autophagy, neuropathies, immunity, viral infection, and tumorigenesis. Genetic profiling has linked USP14 overexpression to many important diseases.^24,25^ Knockout of USP14 has shown beneficial phenotypes, indicating that USP14 is a potential drug target for cancer therapy.^18,25–35^ As tool compounds, IU1 and IU1-47 have been employed in interesting cell-based studies.^25,32,36–38^ However, the relatively weak activity (IC_50_, i.e., μM) of the IU-series compounds has to be improved for the purpose of proof-of-concept studies.

In this study, we reported the co-crystal structure of the catalytic domain of USP14 bound to IU1 and its analogs. Unlike the commonly perceived binding mode in which small-molecule compounds interact directly with the cysteine in the catalytic center, IU1 and its three analogs bind to a site in the thumb-palm cleft region that is distant from the catalytic center. This binding did not trigger in USP14 a conformational change that led to inactivity; instead the binding blocked the access of the C-terminus of ubiquitin to the catalytic center. This proposed mechanism was supported by structure-guided mutation of USP14 and analysis of the structure-activity relationships (SARs) of IU1 analogs.

Our results are especially interesting when we consider the recently reported USP7 inhibitor FT671. USP7, as a well-characterized USP family member, promotes P53 degradation by stabilizing MDM2 at the cellular level via deubiquitination.^39–41^ The activity of USP7 has been correlated to a variety of tumors in several previous studies.^42^ After much effort, a number of new inhibitors towards USP7 have been recently discovered, including FT827, FT671, GNE6440, GNE6776, XL188 and compound 4.^5,8,9,43^ Specifically, the reported binding mode of FT671 to USP7 is consistent with our findings regarding the binding of IU1 to USP14. Superimposition of the USP14-IU1 and USP7-inhibitor complexes showed that IU1 and FT671 occupy similar ubiquitin-binding clefts that lead to the active site in DUBs, preventing the access of the C-terminus of ubiquitin to the catalytic center; IU1 blocks the groove in a vertical direction, while FT671 binds in a horizontal direction (Supplementary information, Fig. S7a, c). The distance between IU1 and the catalytic center was 8.3 Å, which is longer than that observed for FT671 (5.5 Å). The residues that mediate the interactions of these two USP proteins with their inhibitors are apparently different. The USP14 residues H426, Y436, and Y476 participate in π–π stacking with IU1. In contrast, V296 and Q297 of USP7 form hydrogen bonds with FT671 (Supplementary information, Fig. S7b, d). This finding explains why the binding affinity of FT671 is much higher (nM) than that of IU1 with their respective targets.

The results of the comparison of the FT671/USP7 and IU1/USP14 pairs have some important implications in the future design of steric inhibitors of USP14 and other DUBs. Unlike the binding site of FT671 in USP7, the binding site of IU1 in USP14 is snug and is unlikely to accommodate a larger, more elaborate compound.^43^ This result therefore implies that it would be very challenging to include additional nonpolar interactions (e.g., Van der Waals forces, hydrophobic interactions) to improve the binding affinity. Exploration of other ubiquitin binding sites might be necessary and possible, as revealed by the fact that GNE-6776 occupies a different site from FT671 in USP7. Second, maneuvering around the delicate yet strong π–π stacking is likely to be tricky, as reflected by our limited success in the structure-guided optimization of IU1. Even though our best compound (IU1-248) exhibited a 10-fold higher potency than that of IU1, this compound is far from being an ideal tool compound for proof-of-concept studies. The success of FT761 indicates that proper hydrogen bonding is crucial. The binding modes observed in this study suggested some key amino acid residues that one could exploited in future compound design.

Our findings suggest that all the known selective inhibitors of DUBs exert their function via allosteric regulation, not active site inhibition. These allosteric regulators can be classified into two types: (1) allosteric inhibitors, exemplified by XL188 and compound 4; (2) steric blockade inhibitors, exemplified by FT671 and the IU series. Importantly, once the apo structures of other DUBs such as USP2, USP5 (IsoT) and USP15 become available, it would be straightforward to assess whether such a steric site exists in these DUBs. Since both types of inhibition of USP7 have been reported, it might also be possible to capture these DUBs in the autoinhibition mode with a small molecule binding at an allosteric site.

In conclusion, we report that IU1 inhibits USP14 by binding to the thumb-palm cleft region of the USP14 catalytic domain, which prevents the substrate from binding to the enzyme. Our data are consistent with such a steric blockade mechanism and account very well for the selectivity of IU1. The co-crystal structures indicated some important key interactions between IU1 and USP14, including a unique and delicate π-π stacking interaction, which were validated by protein point mutations and inhibitor SAR analysis. Based on this structural analysis and our optimization study, the modification of IU1 for the formation of outer pocket hydrogen bonds is required for successful discovery of a lead-like compound to target USP14. We believe that this intriguing steric blockade mechanism, as also observed in the case of FT671 with USP7, might provide a powerful approach for the discovery of selective DUB inhibitors.

## Materials and methods

### Protein preparation

Glutathione S-transferase (GST)-tagged full-length USP14 and catalytic domain (residues 96–494) of USP14 were cloned into the pGEX-6P-1 vector. All proteins were overexpressed and purified as described previously.^11^ Point mutations were generated by PCR using the QuikChange Site-Directed Mutagenesis Kit (TransGen Biotech, China), and the proteins were overexpressed and purified by the same method.

### Preparation of proteasome-VS

Affinity purification of the 26S human proteasome was carried out as described previously.^7^ Human 26S proteasome was isolated from a stable HEK293T cell line harboring HTBH-tagged hRPN11 (a gift from L. Huang). To prepare proteasome-VS, we incubated 26S proteasome with excess Ub-VS (a gift from the Lei Liu laboratory, Tsinghua University) on the resin for 2 h at room temperature. Unbound Ub-VS was removed by washing the resin with 20 bed volumes of washing buffer. Finally, Ub-AMC hydrolysis assays were carried out to verify the elimination of the DUB activity of proteasome-VS.

### Crystallization of USP14^CAT^ bound to IU1 analogs

Before crystallization, 10 mM IU1 analogs (dissolved in 50% DMSO) were incubated with 250 μM USP14^CAT^ for 1 h at 18 °C. Crystals were grown for 4–7 days at 18 °C by the hanging drop method by mixing the USP14^CAT^-IU1 analogs with an equal volume of reservoir solution containing 18% PEG 3350 (w/v), 450 mM NH_4_F, 100 mM glycine, and 100 mM cesium chloride. Crystals were equilibrated in a cryo-protectant buffer containing reservoir buffer and 20% ethylene glycol (v/v) and were flash frozen in a cold nitrogen stream at –170 °C.

### X-ray data collection and structure determination

Native diffraction data sets for USP14^CAT^-IU1, USP14^CAT^-IU1-47, USP14^CAT^-IU1-206, and USP14^CAT^-IU1-248 were collected on beamline BL17U1 at the Shanghai Synchrotron Radiation Facility and processed using HKL2000.^44^ Subsequent processing was carried out by programs from the CCP4 suite.^45^ The USP14^CAT^-IU1 structure was solved by molecular replacement with PHASER.^46^ The human USP14 catalytic domain (PDB ID: 2AYN)^11^ was selected as the research model for molecular replacement. The USP14^CAT^-IU1 structure was used as the research model for molecular replacement to subsequently determine the other three structures. All structures were refined with PHENIX.^47^ All the final models contained two molecules in each asymmetric unit. There was no clear electron density for residues 96–100, 220–238, 332–338, and 377–399. These residues are likely flexible and disordered in the crystals.

### Ub-AMC hydrolysis assay

Ub-AMC was a gift from the Lei Liu laboratory (Tsinghua University). It was used to measure the deubiquitinating activity of USP14 variants. The deubiquitination assay was performed by following a published protocol.^7^ Briefly, the reaction system contained 50 mM Tris-HCl (pH 7.5), 1 mM EDTA, 1 mg/mL ovalbumin, 5 mM ATP/MgCl_2_ (freshly prepared), 1 mM DTT (freshly prepared), 1 nM Ptsm-VS, and 15 nM USP14; 15 μM IU1 was added to the system if needed. To start the reaction, 1 μM Ub-AMC was added to the system. Ub-AMC hydrolysis was measured at Ex355/Em460 using an EnVision plate reader (PerkinElmer). Fluorescence intensity was recorded every 30 s for 15 min. Each experiment was repeated three times, and the average value was calculated.

### In vitro Ub-PA assay

Ub-PA was a gift from the Lei Liu laboratory (Tsinghua University). For Ub-PA binding, 3 μM DUB protein (USP14^CAT^, USP7^CAT^, UCH-L1, or UCH-L3) was mixed with 12 μM Ub-PA for at least 1 h at room temperature in a reaction system containing 25 mM Tris-HCl (pH 7.5) and 150 mM NaCl. IU1 was preincubated with the DUB protein for 1 h at 25 °C if needed. The DMSO concentration should be no more than 10% (v/v). All the samples were analyzed by SDS-PAGE, and the results are shown.

### Synthesis of compound IU1-248

1-[1-(4-cyanophenyl)-2,5-dimethyl-1H-pyrrol-3-yl]-2-(4-hydroxyl) piperidylethan-1-one (IU1-248). 4-Hydroxypiperidine (0.638 g, 6.30 mmol) was added to a solution of 2-chloro-1-[1-(4-cyanophenyl)-2,5-dimethyl-1H-pyrrol-3-yl]ethan-1-one (1.56 g, 5.73 mmol) and triethylamine (1.59 mL, 11.46 mM) in 30 mL of acetonitrile. The resulting mixture was heated to 85 °C for 2 h. The mixture was concentrated under a vacuum, and the residue was redissolved in 30 mL of ethyl acetate, washed with saturated NaHCO_3_ aqueous solution, dried with anhydrous Na_2_SO_4_ and purified by silica column chromatography to obtain IU1-248 as a white solid (0.93 g, 2.76 mmol) at a 48.2% yield.

*1H NMR* (DMSO-*d*^*6*^, 400 MHz) *δ* 8.05 (d, *J* = 8.3 Hz, 2 H), 7.59 (d, *J* = 8.3 Hz, 2 H), 6.52 (s, 1H), 4.53 (s, 1H), 3.42 (d, *J* = 25.6 Hz, 2H), 2.85–2.63 (m, 2H), 2.23 (s, 3H), 2.16 (t, *J* = 10.1 Hz, 2H), 1.95 (s, 3H), 1.73 (t, *J* = 21.2 Hz, 2H), 1.48–1.29 (m, 2H). *13C NMR* (DMSO-*d6*, 101 MHz) *δ* 193.26, 140.81, 134.84, 133.72, 129.34, 127.97, 119.35, 118.17, 111.56, 108.07, 66.16, 65.50, 51.21, 34.44, 12.60, 12.42.

## Electronic supplementary material

Supplementary information, Fig. S1

Supplementary information, Fig. S2

Supplementary information, Fig. S3

Supplementary information, Fig. S4

Supplementary information, Fig. S5

Supplementary information, Fig. S6

Supplementary information, Fig. S7

## Electronic supplementary material

**Supplementary information** accompanies for this paper at 10.1038/s41422-018-0091-x.
