# Supplementary material for: Small molecule inhibitors reveal allosteric regulation of USP14 via steric blockade
Source: Cell Res. 2018 Sep 25;28(12):1186–94. doi: 10.1038/s41422-018-0091-x (PMC6274642; doi:10.1038/s41422-018-0091-x)
Supplement: Supplementary file 1 — Supplementary information, Fig. S1 [file 41422_2018_91_MOESM1_ESM.pdf]

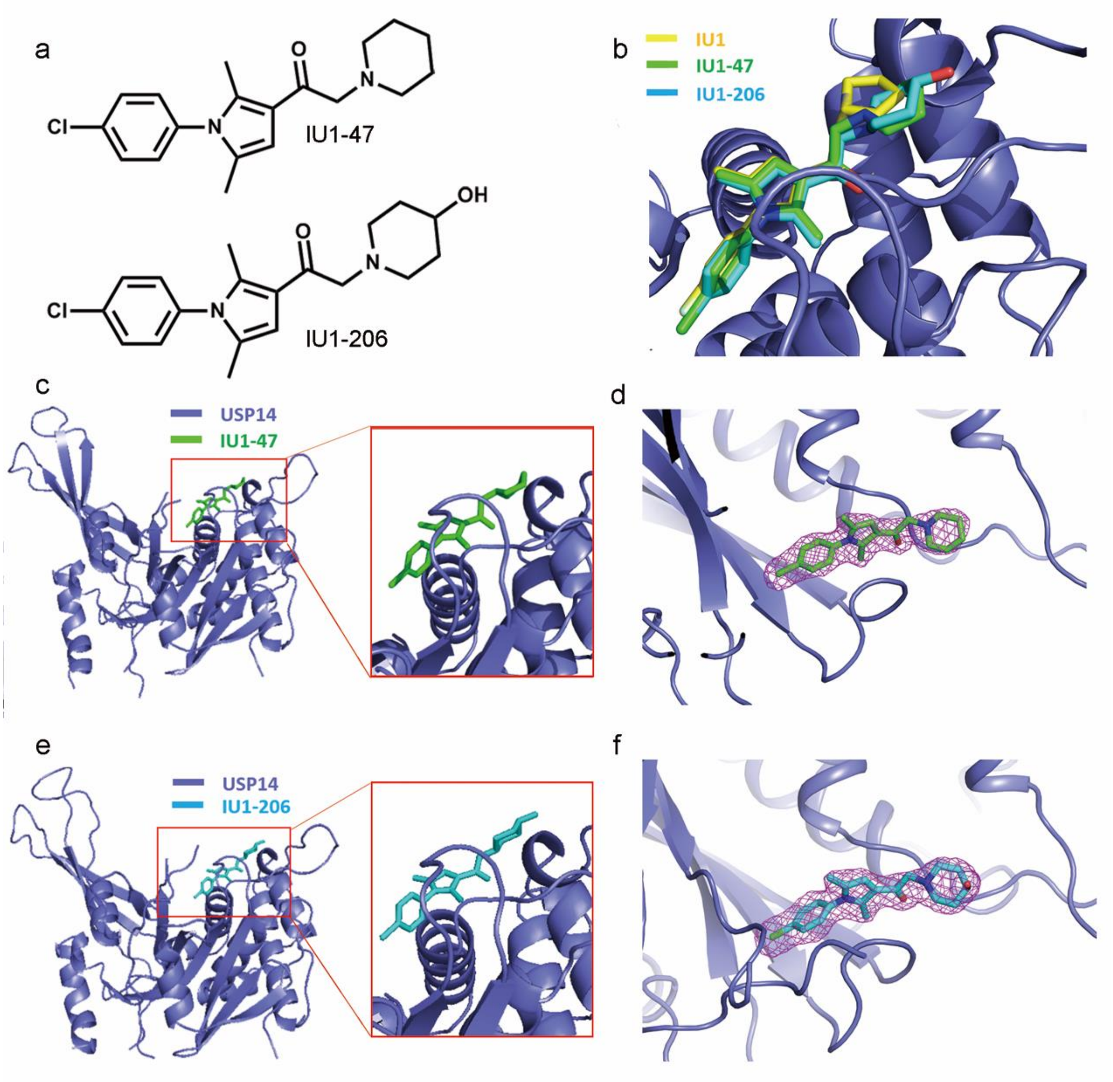

Supplementary information, Fig. S1 Co-crystal structures of USP14^{CAT} bound to IU1-47 and IU1-206.

(a) Chemical structures of IU1-47, and IU1-206. **(b)** Structural comparison of IU1 (yellow), IU1-47 (green) and IU1-206 (cyan) bound to USP14^{CAT} (slate). **(c)** Structure of IU1-47 bound to USP14^{CAT}. **(d)** Stereo representation of the 2|Fo| − |Fc| electron density map of IU1-47, contoured at 1.5σ and covering all atoms. **(e)** Structure of IU1-206 bound to USP14^{CAT}. **(f)** 2|Fo| − |Fc| electron density map of IU1-206, contoured at 1.5 σ and covering all atoms.
