# Supplementary material for: Small molecule inhibitors reveal allosteric regulation of USP14 via steric blockade
Source: Cell Res. 2018 Sep 25;28(12):1186–94. doi: 10.1038/s41422-018-0091-x (PMC6274642; doi:10.1038/s41422-018-0091-x)
Supplement: Supplementary file 2 — Supplementary information, Fig. S2 [file 41422_2018_91_MOESM2_ESM.pdf]

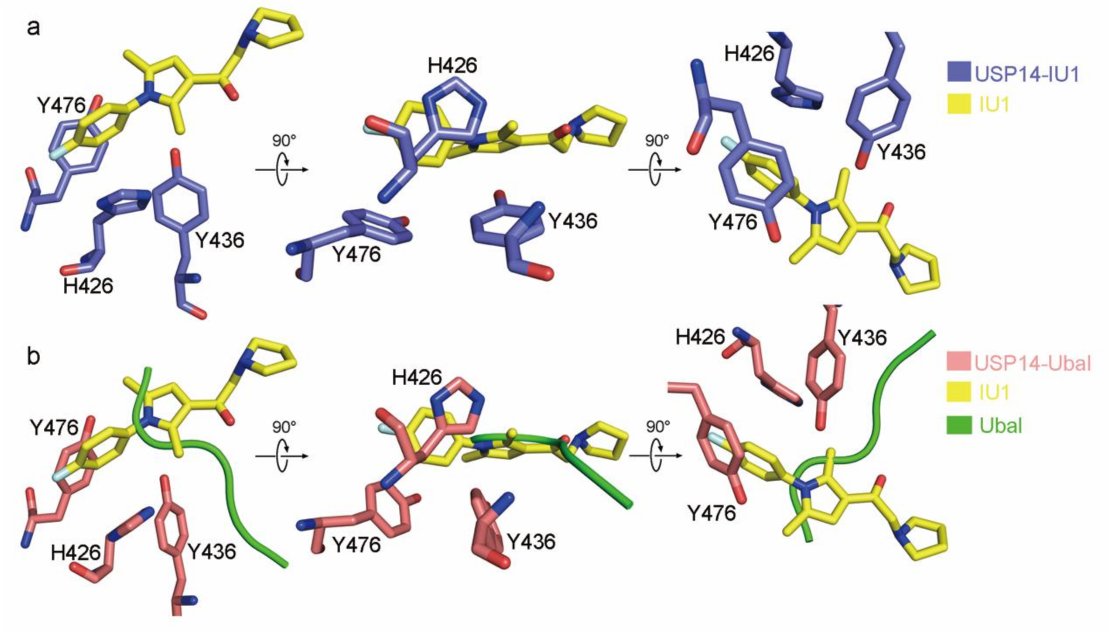

Supplementary information, Fig. S2 Positions of the three key residues H426, Y436 and Y476 in USP14 around IU1.

(a) Positions of H426, Y436 and Y476 in the USP14^{CAT}-IU1 complex. **(b)** Positions of H426, Y436 and Y476 in the USP14^{CAT}-Ubal complex. IU1 was aligned into the structure from USP14^{CAT}-IU1.
