# Supplementary material for: Small molecule inhibitors reveal allosteric regulation of USP14 via steric blockade
Source: Cell Res. 2018 Sep 25;28(12):1186–94. doi: 10.1038/s41422-018-0091-x (PMC6274642; doi:10.1038/s41422-018-0091-x)
Supplement: Supplementary file 3 — Supplementary information, Fig. S3 [file 41422_2018_91_MOESM3_ESM.pdf]

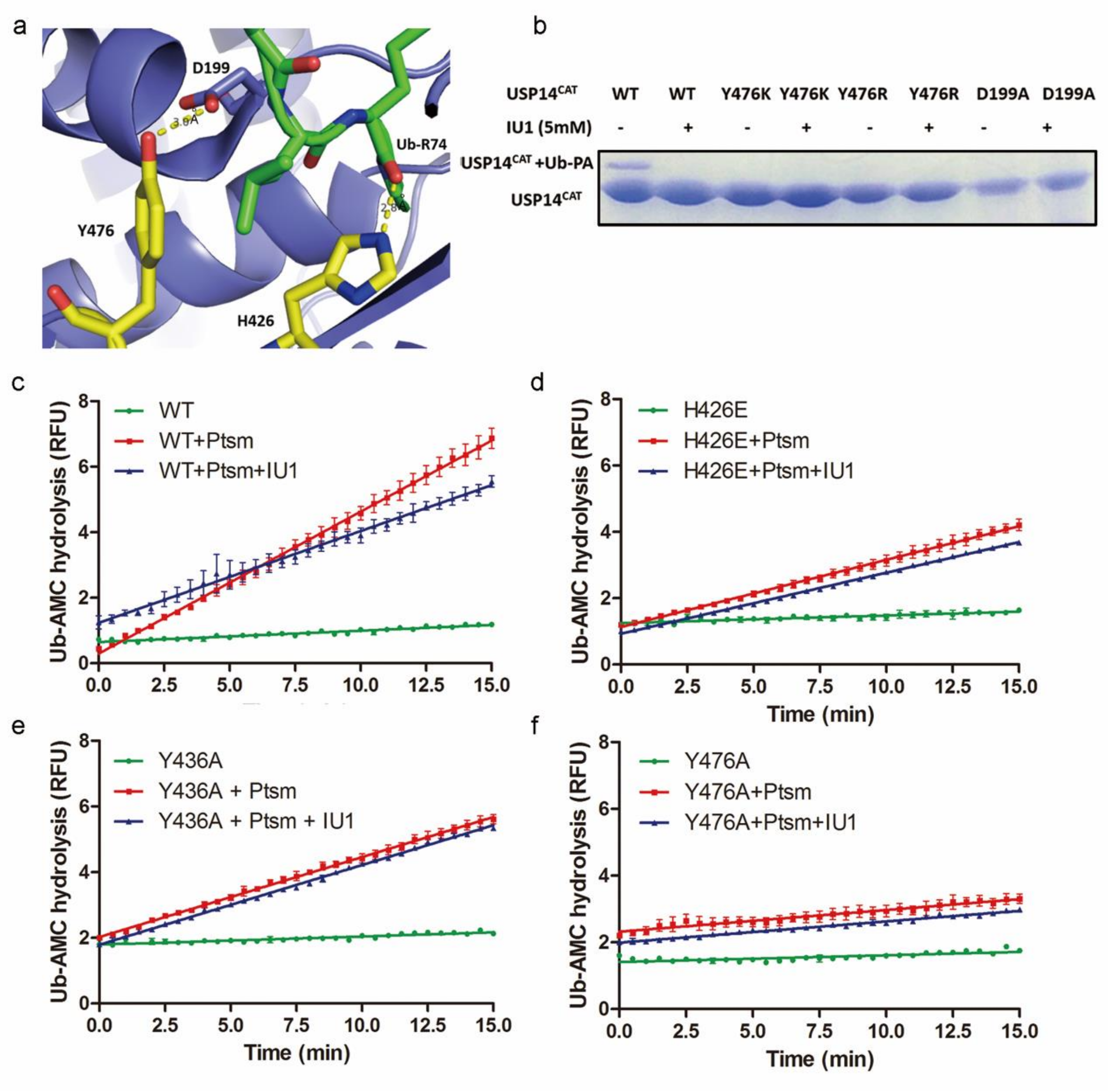

Supplementary information, Fig. S3 Structural and biochemical analyses of the three key residues H426, Y436 and Y476 in IU1 binding.

(a) H426 of USP14 forms a hydrogen bind with R74 of Ub, while Y476 forms a hydrogen bound with D199. **(b)** The Y476K, Y476R and D199A point mutations all abolish the formation of USP14/Ub-PA complex. The result was visualized by SDS-PAGE and Coomassie blue staining. **(c-f)** Linear kinetics of Ub-AMC hydrolysis by USP14 WT, H426E, Y436A and Y476A. All experiments contained 15 nM USP14 and 1 nM proteasome, and all the linear curves have R^2>0.99. Error bars represent SDs.
