# Supplementary material for: Small molecule inhibitors reveal allosteric regulation of USP14 via steric blockade
Source: Cell Res. 2018 Sep 25;28(12):1186–94. doi: 10.1038/s41422-018-0091-x (PMC6274642; doi:10.1038/s41422-018-0091-x)
Supplement: Supplementary file 5 — Supplementary information, Fig. S5 [file 41422_2018_91_MOESM5_ESM.pdf]

a

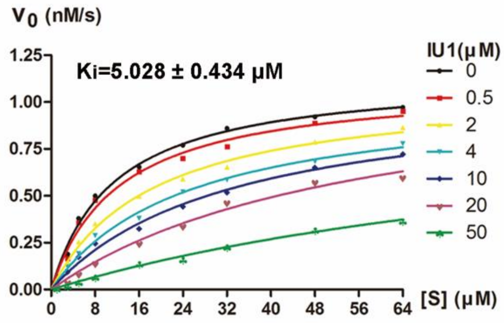


b

| IU1 (μM) | Vm (nM/s) | Km (μM) |
|---|---|---|
| 0 | 1.147 | 11.45 |
| 0.5 | 1.106 | 12.39 |
| 2 | 1.083 | 18.53 |
| 4 | 1.051 | 24.65 |
| 10 | 1.078 | 32.81 |
| 20 | 1.188 | 56.63 |
| 50 | 1.062 | 117.9 |

c

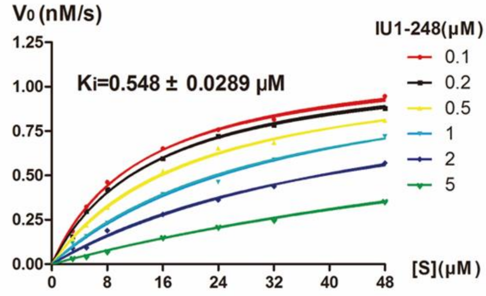


d

| IU1-248 (μM) | Vm (nM/s) | Km (μM) |
|---|---|---|
| 0.1 | 1.2 | 13.79 |
| 0.2 | 1.155 | 14.93 |
| 0.5 | 1.146 | 19.86 |
| 1 | 1.231 | 35.41 |
| 2 | 1.081 | 45.12 |
| 5 | 1.208 | 115.5 |

**Supplementary information, Fig. S5 Kinetic analysis of the inhibitory activity of IU1 and IU1-248 inhibition of USP14 (a)** Michaelis-Menten plot of the concentration-dependence of Ub-AMC (1-64 μM) hydrolysis in the presence of 50 nM USP14 and IU1 (0.5-50 μM). The data were fit to a curve by nonlinear regression ($R^2$>0.99) using the GraphPad Prism program. **(b)** Summary of approximate $V_m$ and $K_m$ values at different concentration of IU1. $K_m$ but not $V_m$ varied with IU1 concentration, indicating that IU1 acts as a competitive inhibitor. **(c)** Michaelis-Menten plot of the concentration-dependence of Ub-AMC (1-48 μM) hydrolysis in the presence of 120 nM USP14 and IU1-248 (0.1-5 μM). The data were fit to a curve by nonlinear regression ($R^2$>0.99) using the GraphPad Prism program. **(d)** Summary of approximate $V_m$ and $K_m$ values at different concentrations of IU1-248. $K_m$ but not $V_m$ varied with IU1-248 concentration, indicating that IU1-248 acts as a competitive inhibitor.
