# Supplementary material for: Small molecule inhibitors reveal allosteric regulation of USP14 via steric blockade
Source: Cell Res. 2018 Sep 25;28(12):1186–94. doi: 10.1038/s41422-018-0091-x (PMC6274642; doi:10.1038/s41422-018-0091-x)
Supplement: Supplementary file 6 — Supplementary information, Fig. S6 [file 41422_2018_91_MOESM6_ESM.pdf]

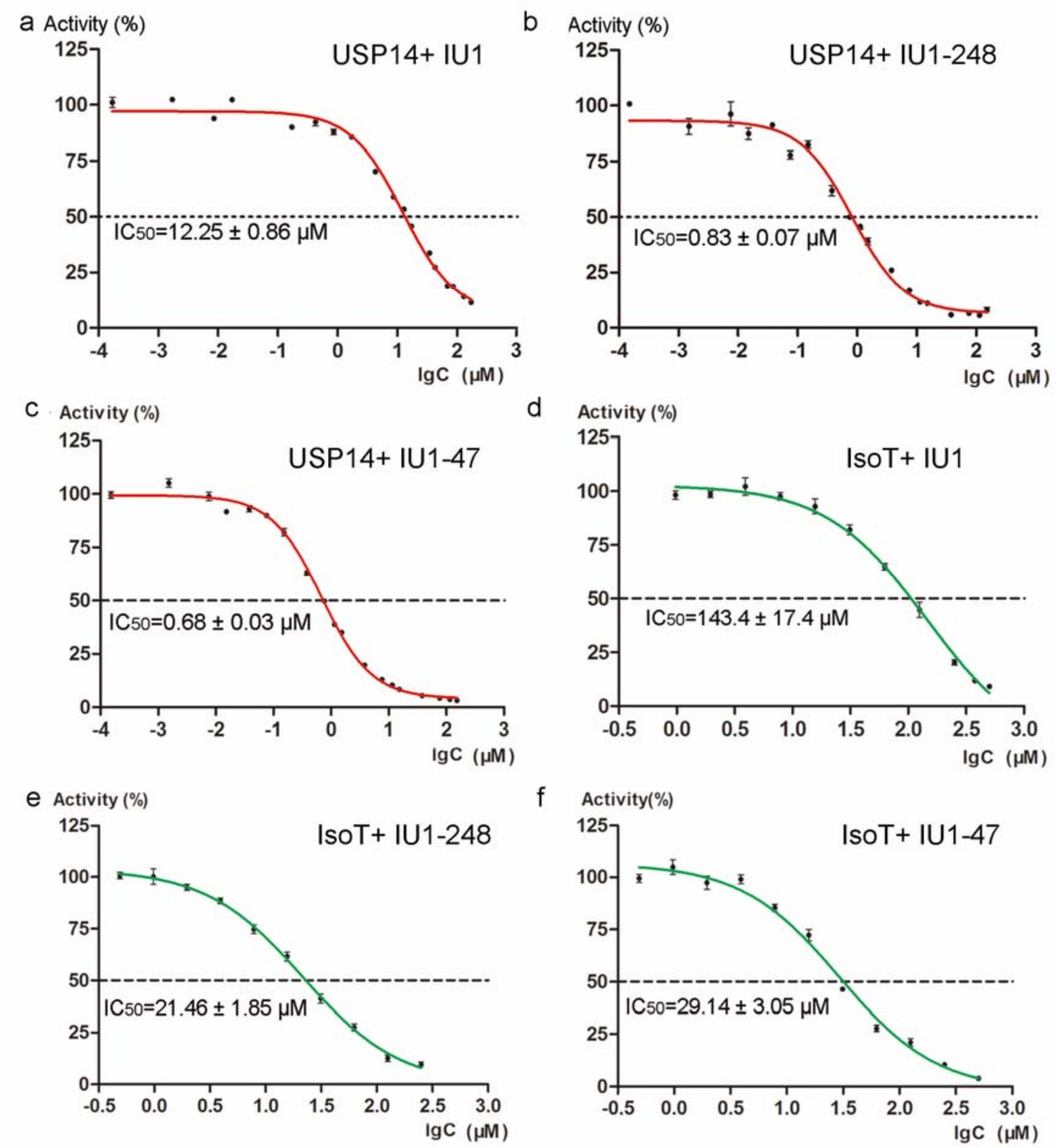

Supplementary information, Fig. S6 Measurement of the IC_{50} of IU1 and its derivatives IU1-248 and IU1-47 towards proteasome-activated USP14 and IsoT. (a-c) Dose-response curves for small-molecule-mediated inhibition of the Ub-AMC hydrolysis activity of proteasome-bound USP14. The reaction system contained 1 μM Ub-AMC, 15 nM USP14 and 1 nM proteasome. **(d-f)** Dose-response curves for small-molecule-mediated inhibition of the Ub-AMC hydrolysis activity of IsoT. The reaction system contained 1 μM Ub-AMC and 10 nM IsoT. All the values correspond to the averages of triplicate experiments. Error bars represent SDs.
