# Supplementary material for: Small molecule inhibitors reveal allosteric regulation of USP14 via steric blockade
Source: Cell Res. 2018 Sep 25;28(12):1186–94. doi: 10.1038/s41422-018-0091-x (PMC6274642; doi:10.1038/s41422-018-0091-x)
Supplement: Supplementary file 7 — Supplementary information, Fig. S7 [file 41422_2018_91_MOESM7_ESM.pdf]

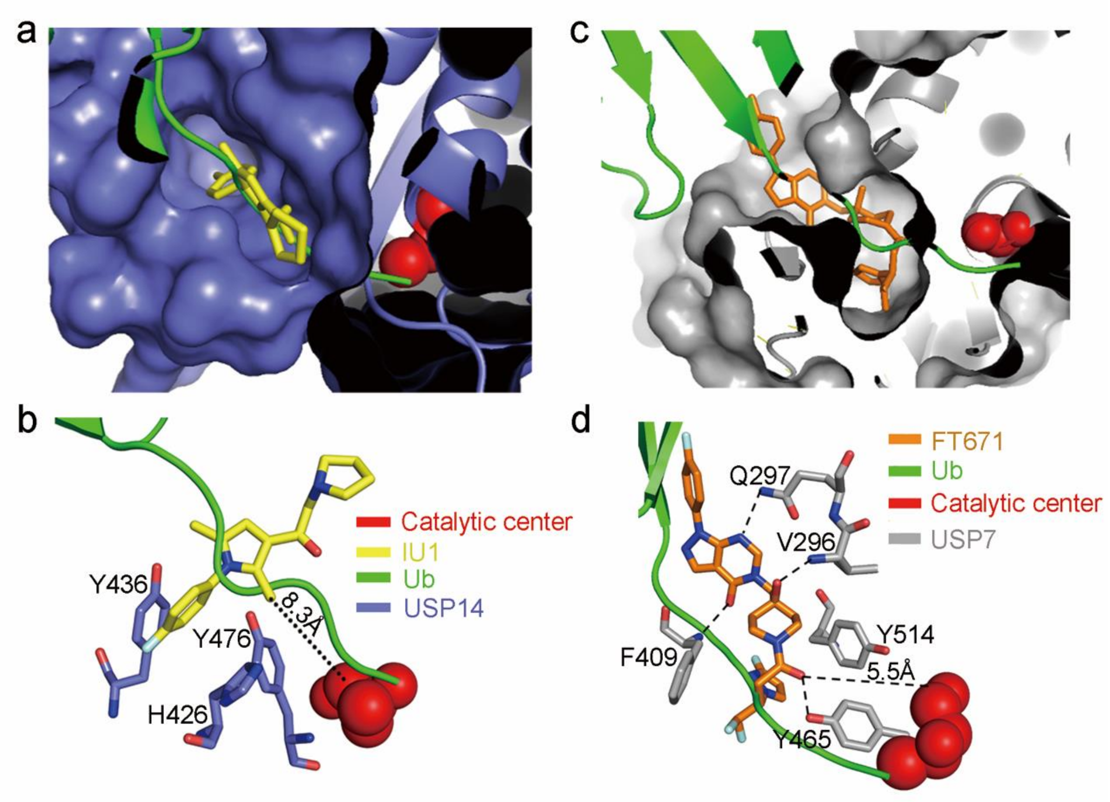


**Supplementary information, Fig. S7 Binding mode comparison of IU1 with FT671. (a-b)** IU1 (yellow) binds outside the cleft that guides the C terminus of ubiquitin (green) into the active site (red), functioning as a barrier. **(c-d)** FT671 (orange) occupies the groove that the C terminus of ubiquitin accesses.
