# Supplementary material for: Small molecule inhibitors reveal allosteric regulation of USP14 via steric blockade
Source: Cell Res. 2018 Sep 25;28(12):1186–94. doi: 10.1038/s41422-018-0091-x (PMC6274642; doi:10.1038/s41422-018-0091-x)
Supplement: Supplementary file 4 — Supplementary information, Fig. S4 [file 41422_2018_91_MOESM4_ESM.pdf]

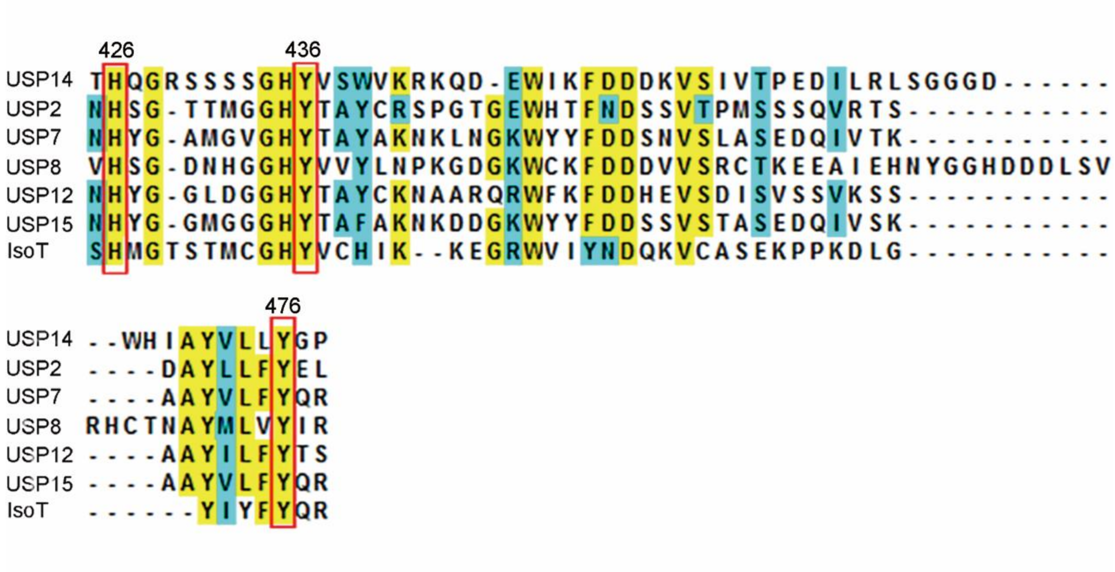

Supplementary information, Fig. S4 Sequence alignment of USP14 with six other USP proteins. The three key residues H426, Y436, and Y476 of USP14 involved in IU1 binding (shown in the red box) are conserved among USP proteins.
